# Special Issue “Viral Shedding and Transmission in Zoonotic Diseases”

**DOI:** 10.3390/v14061190

**Published:** 2022-05-30

**Authors:** Kateri Bertran, Martí Cortey, Miriã F. Criado

**Affiliations:** 1 Unitat mixta d’Investigació IRTA-UAB en Sanitat Animal, Centre de Recerca en Sanitat Animal (CReSA), Campus de la Universitat Autònoma de Barcelona (UAB), 08193 Bellaterra, Spain; 2 IRTA, Programa de Sanitat Animal, Centre de Recerca en Sanitat Animal (CReSA), Campus de la Universitat Autònoma de Barcelona (UAB), 08193 Bellaterra, Spain; 3 Department of Animal Health and Anatomy, Faculty of Veterinary Sciences, Authonomous University of Barcelona, 08193 Barcelona, Spain; 4 Center for Vaccines and Immunology, University of Georgia, Athens, GA 30602, USA

The papers published in this Special Issue represent only a glimpse of the vast diversity of viral infectious diseases, and the complexity of their interactions with the host, that have an impact on human and animal health. Yet, in our opinion, these papers greatly contribute to the scientific knowledge that is crucially needed for their comprehension and control.

In this Special Issue we expanded our knowledge on avian influenza (AI) viruses. We learned that North American duck-origin H7N9 low-pathogenic AI (LPAI) virus infecting chickens can result in polymorphic mutations in multiple gene segments, indicating that waterfowl-origin viruses can readily jump between avian species and spread in poultry populations [1]. We also discovered that mandarin ducks and pigeons play a crucial role in the spread of zoonotic clade 2.3.4.4 H5N6 highly pathogenic AI (HPAI) viruses, which circulate in multiple continents [2]. Molecular characterization of H6N2 LPAI viruses from different countries revealed the importance of genetic surveillance of circulating viruses to improve our knowledge about virus transmission dynamics in natural hosts, virus evolution, and zoonotic potential [3]. Finally, a high-quality, greatly comprehensive review explored the complexities of airborne transmission of H9N2 viruses in mammals, with a special focus on the molecular characteristics of the hemagglutinin [4].

Winged animals, both birds and mammals, were featured for viral diseases other than influenza. We learned that previous exposure to Usutu virus partially protects magpies against lethal challenge with West Nile virus, while still allowing for transmission, with crucial consequences for the ecology of these viruses [5]. Finally, the study of astrovirus infection in a colony of Reunion free-tailed bats suggests that these bats can spill over the virus to other hosts sharing the same habitat, which includes livestock and humans [6].

We would like to acknowledge all the authors that contributed to this Special Issue. By studying the intricacies of viral shedding and transmission of zoonotic viral infectious diseases, we advance in the understanding of their ecology, epidemiology, vaccinology, prevention, and control.
